# Associations Between Cardiovascular Risk Factors and Neurofilament Light Levels Among US Mexican American Adults

**DOI:** 10.1002/brb3.71304

**Published:** 2026-03-12

**Authors:** Monica M. Diaz, Eran Dayan, Health and Aging Brain Study – Health Disparities (HABS‐HD) Study

**Affiliations:** ^1^ Department of Neurology Chapel Hill School of Medicine University of North Carolina Chapel Hill North Carolina USA; ^2^ Department of Radiology and Biomedical Research Imaging Center Chapel Hill School of Medicine University of North Carolina Chapel Hill North Carolina USA

**Keywords:** cardiovascular disease, healthcare disparities, Latinos, Mexican American, Neurofilament light protein, white matter hyperintensities

## Abstract

**Background:**

This study explores the relationship between neurofilament light (NfL) levels, a biomarker of neuronal injury, and cardiovascular health and vascular injury among US Mexican American (MA) and non‐Latino White (NLW) adults.

**Methods:**

Data from 1317 participants in the Health and Aging Brain Study: Health Disparities (HABS‐HD) were analyzed, including phenotypic, neuroimaging, and plasma NfL data. Cardiovascular health factors included hypertension, diabetes, and cardiovascular disease (CVD), while vascular injury markers were white matter hyperintensities (WMH).

**Results:**

We found that NfL levels differed between MA and NLW participants based on diabetes and CVD diagnosis, with more pronounced differences in the MA group. In addition, the association between WMH volume and NfL was steeper in the MA group.

**Conclusion:**

These findings suggest NfL's potential as a prognostic biomarker for CVD and neurodegeneration, especially in MA adults. Further research is needed to understand these associations and develop targeted prevention strategies for brain aging disparities.

## Graphical abstract

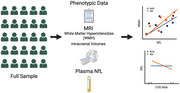

## 1 Introduction

Older Latino adults in the United States are roughly 1.5 times more likely to develop Alzheimer's disease and related dementias (ADRD) compared to non‐Latino White (NLW) adults of similar ages (2023 Alzheimer's Disease Facts and Figures 2023). ADRD prevalence is projected to dramatically increase in the next decades due to changes in life expectancy (Rajan et al. 2021). Given the substantial projected growth of the US Latino community (Matthews et al. 2019), particularly that of US Mexican Americans (MA) (Funk and Lopez 2022), the anticipated impact on ADRD prevalence in this population is alarming. It is thus critical to further investigate the pathophysiological mechanisms that may differentially impact ADRD risk among MA adults compared with NLW in the United States.

A viable strategy for identifying mechanisms that may contribute to differential brain aging outcomes among MA and NLW is via the use of biomarkers. An emerging biomarker for neurodegeneration and axonal injury is the neurofilament light (NfL) protein. Although present in normal aging, NfL levels have also been associated with other neurodegenerative processes, including multiple sclerosis (Domingues et al. 2019; Uher et al. 2020), Parkinson's disease (Mao et al. 2023), and ADRD (Bridel et al. 2019). NfL is a cytoskeletal protein released into cerebrospinal fluid and blood following axonal injury. Unlike neuroimaging markers that demonstrate only macrostructural injury, NfL reflects cumulative neuronal damage that may arise from multiple pathological processes, including small vessel disease, neurodegeneration or inflammation (Peters et al. 2020; Kiani 2025; Loeffler et al. 2020). The literature points to strong associations between NfL and various markers of cardiovasular health and vascular injury. Namely, plasma NfL levels are associated with white matter hyperintensities (WMH) volume (Tang et al. 2024; Chong et al. 2023), an incidental magnetic resonance imaging (MRI) finding, widely considered as reflecting small vessel disease (Wardlaw et al. 2015). Both cross‐sectional and longitudinal studies have demonstrated associations between higher blood NfL levels and greater WMH burden (Peters et al. 2020; Loeffler et al. 2020), as well as subsequent WMH progression and cognitive decline (Tang et al. 2024; Chong et al. 2024), supporting the interpretation of NfL as a dynamic marker of ongoing axonal injury in the context of vascular brain disease. Moreover, higher plasma NfL has also been shown to associate with cardiovascular mortality and CVD (Lohner et al. 2025), and with risk for CVD, coronary heart disease, and heart failure (Aparicio et al. 2022). Plasma NfL levels among Latinos who identify as MA have been reported to associate with CVD risk factors, including hypertension, dyslipidemia and diabetes (O'Bryant et al. 2022). However, it is unknown if the extent to which associations between CVD risk factors and vascular injury markers affect NfL levels differs among Latinos, particularly those who identify as MA.

**FIGURE 1:**
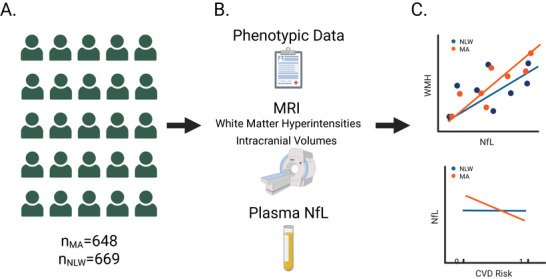
(A) Data from a total of 1317 participants were analyzed, including 648 who self‐identify as Mexican Americans (MA group), and 669 non‐Latino White participants (NLW group). (B) All participants had phenotypic data, MRI data (including white matter hyperintensity and total intracranial volumes) and plasma NfL data. (C) Analyses focused on associations between NfL burden and WMH volume in the MA and NLW groups, and on the contribution of cardiovascular risk factors to NfL burden in these groups.

**FIGURE 2:**
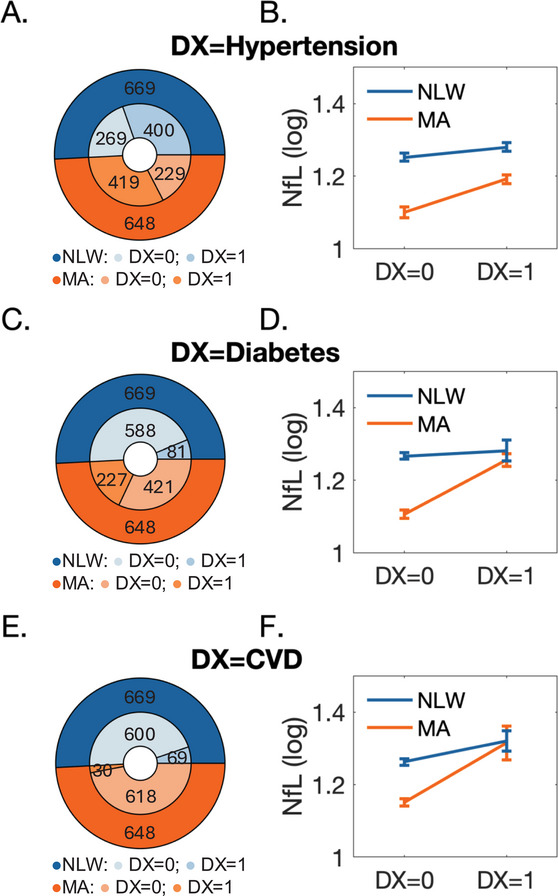
(A) Distribution of hypertension diagnosis did not differ significantly between MA and NLW participants (*p* = 0.068). (B) NfL burden among individuals in the MA and NLW groups did not differ significantly as a function of hypertension (*p* = 0.106, for group × diagnosis interaction). (C) Distributions of diabetes diagnosis differed significantly between the two groups (*p* < 0.001). (D) NfL burden among individuals in the MA and NLW groups differed significantly as a function of diabetes diagnosis (*p* < 0.001, for group × diagnosis interaction). (E) Distributions of cardiovascular disease (CVD) diagnosis were significantly different among the MA and NLW groups (*p* < 0.001). (D) NfL burden among the two groups differed significantly as a function of CVD diagnosis (*p* = 0.007, for group × diagnosis interaction).

The aim of the present study was to examine if associations between plasma NfL levels and markers for cardiovascular health and vascular injury markers differ among US MA and NLW adult participants in the Health and Aging Brain Study: Health Disparities (HABS‐HD). We considered data from a total of 1317 participants (648 MA and 669 NLW; Figure 1A), including phenotypic, neuroimaging, and plasma NfL data (Figure 2B). Cardiovascular health factors included participants’ diagnoses of hypertension, diabetes and CVD, while vascular injury markers included total volume of WMH. We hypothesized that cardiovascular health factors and WMH volumes would differentially impact NfL levels in MA and NLW participants (Figure 1C).

## 2 Methods

### 2.1 Participants

All data were collected as part of the HABS‐HD (O'Bryant et al. 2021), a study initiated by the University of North Texas Health Science Center to investigate the factors underlying health disparities in cognitive aging and dementia among MA. Inclusion criteria in the HABS‐HD study were self‐reported MA or NLW ethnicity, willingness to provide blood samples, ability to undergo neuroimaging, age 50 and older, and fluency in English or Spanish. Exclusion criteria were type 1 diabetes, active infections, current or recent (<12 months) cancer other than skin cancer, current diagnosis of severe mental illness that can impact cognition (excluding depression), recent (<12 months) traumatic brain injury with loss of consciousness, current or recent substance abuse, active medical conditions that can impact cognition, and current diagnosis of non‐AD dementia.

Here we considered baseline, cross‐sectional data from participants who self‐reported MA (*n* = 648) or NLW ethnicity (*n* = 669) and had available neuroimaging (WMH volume and intracranial volume [ICV]). and plasma NfL data. All subjects provided written informed consent, and study procedures were all approved by the local Institutional Review Board.

### 2.2 Clinical Definitions

Utilizing previously established procedures by HABS‐HD, diabetes was defined as A1C ≥ 6.5 OR (Rajan et al. 2021) past medical history of diabetes. CVD was defined as hypertension (yes, no), CVD (e.g., heart attack, heart failure, cardiomyopathy, atrial fibrillation, and/or heart valve replacement; yes, no). Per HABS‐HD procedures, all medical history (including self‐reported medication use) is reviewed by a medical professional associated with HABS‐HD and is used to assist in the determination of comorbid medical conditions, including CVD and diabetes. Medical comorbid factors are determined based on (1) self‐reported medical history, (2) clinical labs, (3) medication list, and (4) objective measures (O'Bryant et al. 2021).

### 2.3 Neuroimaging Data Acquisition and Metric Extraction

Multimodal MRI data were acquired from participants using a 3T Siemens Magnetom SKYRA scanner. Here, we analyzed data acquired with 3D T1‐weighted whole brain magnetization prepared rapid acquisition gradient echo (MPRAGE) (1.1 × 1.1 × 1.2 mm; TR = 2300 ms; TE = 2.93 ms) and fluid attenuated inversion recovery (FLAIR) (1.0 × 1.0 × 1.2 mm; TR = 4800 ms; TE = 441 ms) sequences. Total volumes of WMH were estimated using FLAIR and MPRAGE data by the HABS‐HD team (King et al. 2022) with the Lesion Growth Algorithm (Schmidt et al. 2012), available in the Lesion Segmentation Toolbox in the Statistical Parametric Mapping (SPM) software suite. ICVs and hippocampal volumes (left, right) were estimated for each participant using the segmentation pipeline (Fischl et al. 2002) in FreeSurfer 6.0 and based on MPRAGE data. Total WMH, hippocampal volumes, and ICV were all obtained from the HABS‐HD dataset as derived variables.

### 2.4 Blood Sample Collection and Processing

Procedures for blood collection and processing were described elsewhere (O'Bryant et al. 2021, 2022). Briefly, fasting blood samples were collected and processed within 2 h post‐collection, in accordance with established guidelines (Osborn et al. 2019). Plasma NfL was assayed from participants’ samples using the Quanterix single molecule array (Simoa HD1) platform.

### 2.5 Socioeconomic and Sociocultural Factors

We also assessed the contribution of socioeconomic and sociocultural factors to the associations queried in this study. Socioeconomic and sociocultural variables analyzed included household income and acculturation, assessed with the Short Acculturation Scale for Hispanics (SASH) (Marin et al. 1987).

### 2.6 Statistical Analysis

All statistical analyses were performed with JASP 0.16.4 and MATLAB R2023.a. Pairwise differences in demographic, imaging or biomarker data were tested with independent sample *t* tests or Mann–Whitney U tests (used when Gaussian distributions could not be assumed). Categorical variables were compared with a chi‐squared test. Correlations between variables were examined with the Pearson correlation coefficient (*r*). Continuous variables with non‐ Gaussian distributions (NfL and WMH) were log‐transformed in all correlations analyses. The impact of three CVD risk factors we consider here (hypertension, diabetes, diagnosis of CVD) on NfL burden was examined with Analysis of Covariance (ANCOVA) models, where NfL burden served as the dependent factor, and sex, group (MA, NLW), and each CVD risk variable served as factors. Given that our planned analyses were centered on the contribution of a factor (ethnoracial group) to the associations between two continuous variables (one covariate of interest and one dependent variable), and given the strong correlation between outcomes and covariates, ANCOVA was performed. Normal distributions were assumed for continuous variables or approximated via log transformation. The models were further adjusted for age and education. To examine if ethnicity differentially impacted the association between WMH and NfL, we performed an ANCOVA where WMH volume served as the dependent variable, group and sex as factors and NfL as a covariate of interest. The model was further adjusted for age, education, and ICV. Continuous variables with non‐Gaussian distributions (NfL and WMH volume) were log‐transformed in the ANCOVA model. Separate follow‐up analyses evaluated the potential effect of creatinine levels and body mass index (BMI) and of household income and acculturation on the reported results by including these variables as covariates in the ANCOVA models described above.

## 3 Results

### 3.1 Demographics

Data from a total of 1317 participants were analyzed (Figure 1), including 648 who self‐identified as MA and 669 who identified as NLW. Male‐to‐female distributions differed between the two groups (*χ* ^2^ = 23.776, *p* < 0.001), with a larger proportion of males in the MA group. Participants in the MA group were also younger (*t*_1315_ = 12.755, *p* < 0.001), had lower educational attainment (*t*_1315_ = 28.621, *p* < 0.001), lower total ICV volumes (*t*_1315_ = 12.118, *p* < 0.001), lower WMH volumes (Mann–Whitney U test, *p* < 0.001), and lower NfL burden (Mann–Whitney U test, *p* < 0.001) (Table 1).

**TABLE 1:** Descriptive and inferential statistics of demographics and study outcomes in the study sample.

	MA (*n* = 648)		NLW (*n* = 669)				
	*M*	SD	*M*	SD	*t*	*p*	*d*
Demographic variables							
Age	63.30	8.10	69.22	8.72	12.76	<0.001	0.703
Education (years)	9.63	4.65	15.50	2.52	28.62	<0.001	1.578
Study outcomes							
ICV	1.35 × 10 + 6	156699.76	1.46 × 10 + 6	187338.16	12.12	<0.001	0.668
WMH volume	2.44	4.94	4.05	7.46		<0.001	
NfL	17.25	13.18	21.23	14.10		<0.001	

Abbreviations: ICV, intracranial volume; MA, Mexican American; MRI, magnetic resonance imaging; NFL, neurofilament light; NLW, non‐Latino White; WMH, white matter hyperintensity.

### 3.2 Impact of Cardiovascular Health Risk Factors on NfL Burden

We first examined whether ethnicity interacted with cardiovascular health risk factors and their impact on NfL burden (dependent variable). This analysis considered three common cardiovascular health risk factors: Hypertension, diabetes, and diagnosis of CVD. Rates of hypertension (MA: 419 of 648 participants [64.7%]; NLW: 400 of 669 participants [59.8%]) in the sample analyzed here did not differ between the MA and NLW groups (*χ* ^2^ = 3.32, *p* = 0.068) (Figure 2A). Moreover, NfL burden in MA and NLW participants did not differ significantly between individuals with or without a diagnosis of hypertension (group × diagnosis interaction: *F*_1,1310_ = 2.61, *p* = 0.106) (Figure 2B, Table S1).

Different results were observed for diabetes. Rates of diabetes diagnosis (MA: 227 of 648 participants [35%]; NLW: 81 of 669 participants [12.1%]) differed among the two groups (*χ* ^2^ = 96.538, *p* ≤ 0.001) (Figure 2C), with higher rates seen among MA participants. Moreover, NfL burden levels among MA and NLW participants differed as a function of diabetes diagnosis (group × diagnosis interaction: *F*_1,1310_ = 14.692, *p* ≤ 0.001). Namely, differences between participants with or without diabetes, were steeper in the MA group (Figure 2D, Table S2). When we added creatinine (mg/dL) and BMI (kg/m^2^) as covariates, the interaction remained significant (*F*_1,1296_ = 7.961, *p* = 0.005), but the strength of the association decreased somewhat (see Tables of Supporting Information).

Diagnosis of CVD (MA: 30 of 648 participants [4.6%]; NLW: 69 of 669 participants [10.3%]) also differed between the two groups (*χ* ^2^ = 15.299, *p* ≤ 0.001), with higher rates seen in the NLW group (Figure 2E). However, as in diabetes, differences among individuals with and without CVD diagnosis were steeper in the MA group (group × diagnosis interaction: *F*_1,1310_ = 7.211, *p* = 0.007) (Figure 2F, Table S3). When we added creatinine (mg/dL) and BMI (kg/m^2^) as covariates, the association remained significant (*F*_1,1296_ = 11.102, *p* ≤ 0.001), increasing in strength relative to the original interaction. Including creatinine (mg/dL) and BMI (kg/m^2^) as covariates in the model, given their potential impact on associations between cardiovascular health risk factors and NfL levels, did not affect the strength of the interaction between ethnicity and hypertension diagnosis (*F*_1,1296_ = 1.195, *p* = 0.275) (see Tables of Supporting Information).

Finally, to account for comorbidities between the cardiovascular health risk factors considered here, we repeated all ANCOVA models listed above for each diagnostic label (hypertension, diabetes, CVD), adjusting each time for the other two diagnoses. Similar group × diagnosis interactions were obtained in each of the three models (Table S4).

As a follow‐up analysis, we also evaluated whether any of the reported ethnicity by cardiovascular health risk factors interactions changed when adjusting for socioeconomic and sociocultural factors, including SASH scores (measure of acculturation and annual income). All significant interactions were retained when adjusting for these factors (see Tables of Supporting Information).

### 3.3 Impact of Ethnicity on Association Between Vascular Injury Markers (WMH Volume) and NfL

**FIGURE 3:**
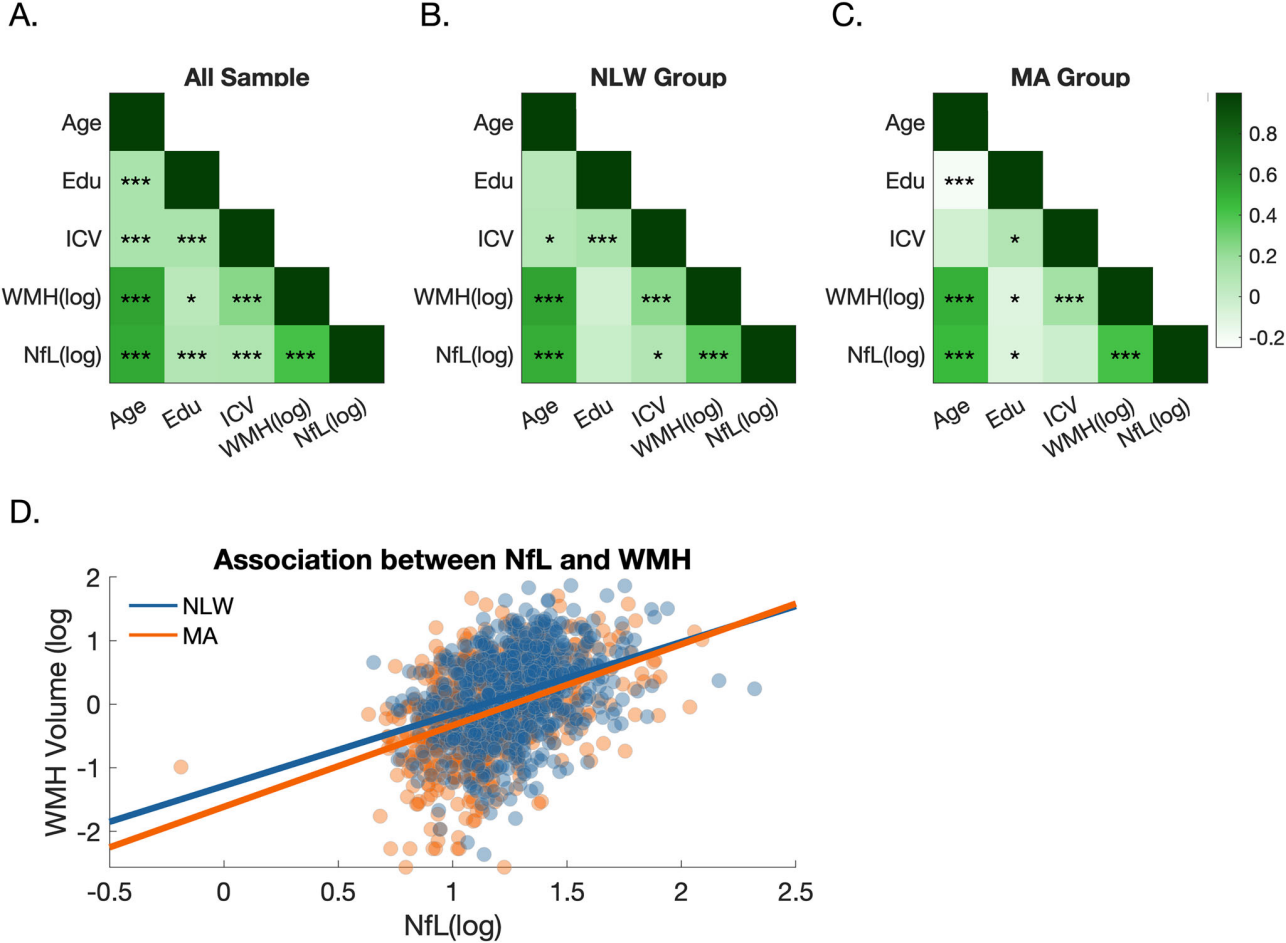
(A) Associations (Pearson's correlations) between age, education, ICV, total WMH volume (log‐transformed) and NfL burden (log‐transformed) are shown for the entire sample and for the NLW (B) and MA (C) groups separately. (D) The association between NfL burden (log‐transformed) and WMH volume (log‐transformed) differed significantly between individuals in the MA and NLW groups (*p* = 0.003, for group × NfL interaction, adjusting for age, sex, ICV and education), with a steeper association observed for individuals in the MA group. *p< 0.05, **p < 0.01, ***p < 0.001.

We next set out to examine if NfL levels among MA and NLW participants were differentially associated with WMH volume, a widely used marker for small vessel disease in the brain. First, we estimated associations between WMH volume, NfL, and the background demographic variables considered here (age and education). Associations between each variable and total ICV were examined as well, since WMH volume is likely to covary with this variable. In all analyses, NfL and WMH volume were log‐transformed to adjust for their skewed distributions. When considering the entire sample (Figure 3A), strong correlations were observed between NfL and WMH volume (*r* = 0.416, *p* < 0.001), and between these two variables and age, education and total ICV (all *p* values ≤ 0.04). Correlations were generally similar when data from each group was considered separately (Figure 3B,C), with a higher correlation observed between WMH and NfL in the MA group (*r* = 0.422, *p* < 0.001), compared to the NLW group (*r* = 0.351, *p* < 0.001). We thus next compared the strength of these association more directly with an ANCOVA model. This analysis revealed that the association between WMH volume and NfL (dependent variable) varied between the two groups (group × WMH interaction: *F*_1,1298_ = 8.791, *p* = 0.003), with a steeper association observed in the MA group (Figure 3D, Table S5). When we added creatinine (mg/dL) and BMI (kg/m^2^) as covariates, the interaction remained significant (*F*_1,1285_ = 5.406, *p* = 0.02), but decreased somewhat in strength (see Tables of Supporting Information).

To test if the results were specific to NfL, we conducted additional sub‐analyses and replaced NfL (dependent variable) with right, left, and total hippocampal volumes in ANCOVA models testing, if the association between WMH volume and hippocampal volume (dependent variable) varied as a function of ethnicity. We found that the interactions between WMH and ethnoracial group were not significant for neither right (*F*_1,1298_ = 0.461, *p* = 0.497), nor left (*F*_1,1298_ = 0.128, *p* = 0.721), nor total hippocampal volume (*F*_1,1298_ = 0.011, *p* = 0.917). Therefore the results reported here are specific to NfL (see Tables of Supporting Information).

## 4 Discussion

The study examined the potential impact of ethnoracial background on associations between NfL levels and cardiovascular health and vascular injury factors among adults enrolled in the HABS‐HD cohort. We found that the existence of cardiovascular health risk factors (particularly diagnosis of diabetes and CVD) impacted NfL levels differently when comparing MA and NLW participants, with a larger impact seen among the MA group. We also found differential associations between WMH volume and plasma NfL levels among MA and NLW participants, wherein the MA group displayed a steeper association than the NLW group.

The association between WMH volume and serum NfL has been demonstrated in previous studies, but the effect of ethnoracial background (comparing Latinos vs. NLW) on this relationship has not been studied. For example, significant interaction between ethnoracial group and cardiovascular risk (based on the Framingham risk score) were found for plasma NfL, with a stronger relationship among the MA group, as in the current study (Jiang et al. 2023). Moreover, one prior study found that both NfL and WMH volume were significantly independently associated with cognitive decline, with larger WMH volumes influencing cognitive decline rates the most among those with lower NfL levels (Dhana et al. 2023). Another study found an association between higher plasma NfL and WMH volume, but not with global cognitive impairment (Tang et al. 2024), suggesting that NfL may be a marker of WMH volume but not necessarily cognitive impairment. Similar relationships have been investigated among other ethnoracial groups, such as in Asian cohorts, where high plasma NfL was associated with increased cerebrovascular disease risk and progression (Chong et al. 2023, 2024; Qu et al. 2021). However, some studies did not find significant relationships between WMH and plasma NfL (Hermesdorf et al. 2024), highlighting the need for further investigation in larger cohorts and across different ethnoracial backgrounds. In our data, we found that baseline NfL levels tended to be lower among MA participants compared to NLW for those without a diagnosis of diabetes or CVD (Figure 2), yet those MA who had a diagnosis of diabetes or CVD had higher NfL levels. This may demonstrate that the cumulative effect of CVD or diabetes may have a stronger impact on neurodegeneration among MA compared to NLW.

Our study is, to the best of our knowledge, the first to study the relationship between NfL levels and WMH volumes as a function of ethnoracial background, specifically comparing MA and NLW adults. The HABS‐HD group found that a higher Framingham Risk Score (indicating higher CVD burden) was associated with higher NfL levels only among MA adults and not among NLW. However, this relationship with WMH volume was not explored. Similar to our study, hypertension was associated with NfL in both groups (Jiang et al. 2023), but the degree of the association on MA compared with NLW was not studied at the time.

Our results identify relationships between CVD and NfL, as well as diabetes and NfL, with a stronger impact among MA compared to NLW. Previous studies have demonstrated the association between CVD, including hypertension and cardiovascular death and stroke/transient ischemic attack, and higher serum NfL (Liu and Zhang 2024; Amrein et al. 2023; Hoyer‐Kimura et al. 2021), suggesting its utility as a predictive marker for cardiovascular outcomes. Our findings indicate that these associations may be stronger for MA, highlighting the potential utility of using NfL in this population. Factors including social determinants of health, such as healthcare access and utilization and socioeconomic factors may also have a role in the relationships between ethnicity, CVD/hypertension/diabetes, and neurodegeneration which should also be considered in future research (Galvin et al. 2021).

Several mechanisms may explain why cardiovascular risk factors are more strongly associated with neurodegeneration among MA participants. Cardiovascular risk, social and structural determinants of health may modify the biological consequences of these risks by impacting lifelong exposure to chronic stress, access to preventive care, and cumulative cardiometabolic burden (Acquah et al. 2023; Havranek et al. 2015). Chronic psychosocial stress and socioeconomic disadvantage have been associated with greater systemic inflammation (Muscatell et al. 2020; Stringhini et al. 2013), endothelial dysfunction (Kershaw et al. 2017), and dysregulation of stress‐response pathways (Lê‐Scherban et al. 2018; Zhang et al. 2024), which may exacerbate microvascular brain injury and reduce neuronal resilience (Zhang et al. 2024). In this context, similar levels of cardiovascular risk may lead to disproportionate white matter injury and neuroaxonal damage (Austin et al. 2022; Vriend et al. 2025), reflected by higher plasma NfL levels. These processes demonstrate that social and structural exposures interact with vascular risk to accelerate neurodegeneration. While our study cannot directly test these mechanisms, the observed differential associations between NfL and cardiovascular health as well as markers of vascular injury highlight the importance of identifying these modifiable pathways that may be targeted to reduce ethnoracial disparities in brain aging and dementia risk.

Plasma NfL levels have been previously described in MA populations in several cohorts. Using the HABS‐HD data, one study found that higher NfL was associated with global cognitive decline but was not associated with increased incident dementia risk among MA (Gonzales et al. 2022). We found NHW participants often show higher NfL than MAs among cognitively unimpaired and MCI groups, with group differences attenuating in dementia, supporting the need for ethnoracially‐tailored cut points of NfL levels (Hall et al. 2022). Higher NfL has been associated with worse performance across multiple cognitive domains among MA in the HABS‐HD dataset highlighting its importance in relation to cognition in this population (Hall et al. 2020). Recent HABS‐HD analyses also show that higher hemoglobin A1c is associated with higher NfL among MAs, suggesting that metabolic risk may differentially influence NfL across groups (Yu et al. 2024). These studies point to the importance of utilizing NfL as a marker of neurodegeneration among MA and join the current results in demonstrating the influence of cardiovascular and cardiometabolic factors on NfL in this population.

Our findings should be interpreted within a multimodal framework that recognizes both the strengths and limitations of plasma NfL as a marker of neurodegeneration. WMH quantify macrostructural manifestations of cerebral small vessel disease, while other MRI markers, such as diffusion tensor imaging metrics and regional brain atrophy, capture microstructural white matter integrity and tissue loss, respectively (Duering et al. 2023). In contrast, NfL is a blood‐based, integrative marker of neuroaxonal injury that is sensitive to a range of upstream insults, including vascular injury, neurodegenerative pathology, and inflammatory processes (Gaetani et al. 2019). Our findings align with longitudinal evidence demonstrating that plasma NfL is associated with baseline WMH and may also predict future WMH burden (Sun et al. 2020). Moreover, studies in community samples have shown associations between higher plasma NfL and greater WMH volumes, even after adjusting for major vascular and demographic risk factors (Tang et al. 2024). Therefore, combining NfL with neuroimaging markers of small vessel disease, such as WMH volume, and markers of neurodegeneration may inform pathways linking cardiovascular risk to neuronal injury and to identify those at the highest risk of vascular‐related neurodegeneration.

This study has several limitations worth noting. First, it focuses on a US MA cohort, which restricts the applicability of our findings to other Latino groups. Second, the cross‐sectional observational design prevents us from identifying causal relationships. Moreover, our data did not capture important social determinants of health, such as healthcare access and utilization or socioeconomic status, which may mediate the relationship between ethnicity, cardiovascular risk factors, and neurodegeneration. Future studies should incorporate these important data into their analyses. Additional factors related to WMH volume and NfL burden, such as other cardiovascular risk factors or social and cultural factors, may partly explain the observed differences in white matter changes and NfL burden between MA and NLW groups. In addition, our study did not consider cognitive impairment or decline as an outcome, which has been shown to differentially associate with WMH volume among MA and NLW (Graves et al. 2024). Whether the associations reported here between NfL and cardiovascular health further impact cognitive outcomes should be explored in larger studies, along with mechanisms of reserve and resilience (Langella et al. 2021; Stanford et al. 2022) that may mitigate detrimental impact on cognitive outcomes. We did not report outcomes on cognitive status, given our primary objective was to determine the influence of ethnicity and cardiovascular risk factors on NfL as a surrogate for neurodegeneration. Future studies may assess cognition as a primary clinical outcome to determine how these relationships may influence clinical practice. It is also important to mention that many white matter diseases (Schiffmann and van der Knaap 2009) may not be fully captured via quantification of metrics such as WMH volume. Additional indices capturing white matter disease should be integrated in future research focusing on more diverse study populations. Moreover, the HABS‐HD dataset has the most data available on MA and NLW which is why we selected these two groups. This dataset now includes data on other racial/ethnic groups. In the future, comparison of our findings to those of Black/African Americans or non‐Latino Asian groups would be indicated.

In summary, our findings indicate that the relationship between NfL burden and markers for cardiovascular health varies across ethnoracial groups, with a stronger association observed among MA compared to NLW. These results have significant clinical implications. Specifically, for patients diagnosed with cerebral white matter disease, encouraging modifiable lifestyle changes may enhance cardiovascular health and vascular injury and subsequently reduce neurodegeneration, particularly in MA. Further research involving diverse Latino samples, interventional designs, longitudinal observations, comprehensive examination of cardiovascular health and sociocultural risk factors, and use of other white matter indices is necessary to deepen our understanding of the connections between WMH, cardiovascular health risk factors, and neurodegeneration in MA and other Latino groups at high risk for dementia.

## Author Contributions

Monica M. Diaz: drafting of original draft, methodology, editing of final draft. Eran Dayan: statistical analyses, methodology, editing of final draft.

## Funding

MMD is supported by the NIH NIMH (K23MH131466),

## Consent

All human subjects provided informed consent.

## Conflicts of Interest

The authors declare no conflicts to interest.

## Supporting information




**Supplementary Material**: brb371304‐sup‐0001‐SuppMat.docx

## Data Availability

All data are available online at: https://apps.unthsc.edu/itr/habs‐hd
