# Supplementary material for: Associations Between Cardiovascular Risk Factors and Neurofilament Light Levels Among US Mexican American Adults
Source: Brain Behav. 2026 Mar 12;16(3):e71304. doi: 10.1002/brb3.71304 (PMC13093513; doi:10.1002/brb3.71304)
Supplement: Supplementary file 1 — Supplementary Material: brb371304‐sup‐0001‐SuppMat.docx [file BRB3-16-e71304-s001.docx]

Supplementary Materials for:

**Associations between Cardiovascular Risk Factors and Neurofilament Light Levels Among U.S. Mexican American Adults**

**Authors**: Monica M. Diaz (1), Eran Dayan (2)* for the Health and Aging Brain Study: Health Disparities (HABS-HD) Team**

**Affiliations:**

(1) Department of Neurology, University of North Carolina at Chapel Hill School of Medicine, Chapel Hill, NC USA; [monica.diaz@neurology.unc.edu](mailto:monica.diaz@neurology.unc.edu)

(2) Department of Radiology and Biomedical Research Imaging Center, University of North Carolina at Chapel Hill School of Medicine, Chapel Hill, NC USA; [eran_dayan@med.unc.edu](mailto:eran_dayan@med.unc.edu)

******HABS-HD MPIs: Sid E O’Bryant, Kristine Yaffe, Arthur Toga, Robert Rissman, & Leigh Johnson; and the HABS-HD Investigators: Meredith Braskie, Kevin King, James R Hall, Melissa Petersen, Raymond Palmer, Robert Barber, Yonggang Shi, Fan Zhang, Rajesh Nandy, Roderick McColl, David Mason, Bradley Christian, Nicole Philips and Stephanie Large.

Supplemental Table 1. *Inferential statistics associated with ethnoracial background* × *hypertension interaction effects on individual neurofilament light levels*

| Variables | *F* | df | *p* |
| --- | --- | --- | --- |
| Ethnicity × Hypertension diagnosis | 2.61 | 1 | .106 |
| Main Effects |  |  |  |
| Hypertension diagnosis | 2.57 | 1 | .109 |
| Age | 358.87 | 1 | <.001 |
| Educational level | 0.44 | 1 | .508 |
| Sex | 0.007 | 1 | .934 |
| Ethnicity | 8.7 | 1 | .003 |
| Residuals |  | 1310 |  |
|  | | | |

Supplemental Table 2. *Inferential statistics associated with ethnoracial background* × *diabetes interaction effects on individual neurofilament light levels*

| Variables | *F* | df | *p* |
| --- | --- | --- | --- |
| Ethnicity × Diabetes diagnosis | 14.69 | 1 | <.001 |
| Main Effects |  |  |  |
| Diabetes diagnosis | 22.52 | 1 | <.001 |
| Age | 377.21 | 1 | <.001 |
| Educational level | 0.02 | 1 | .877 |
| Sex | 0.08 | 1 | .783 |
| Ethnicity | 1.27 | 1 | .261 |
| Residuals |  | 1310 |  |
|  | | | |

Supplemental Table 3. *Inferential statistics associated with ethnoracial background* × *cardiovascular disease interaction effects on individual neurofilament light levels*

| Variables | *F* | df | *p* |
| --- | --- | --- | --- |
| Ethnicity × CVD diagnosis | 7.21 | 1 | .007 |
| Main Effects |  |  |  |
| CVD diagnosis | 6.66 | 1 | .010 |
| Age | 371.92 | 1 | <.001 |
| Educational level | 1.06 | 1 | .304 |
| Sex | .003 | 1 | .960 |
| Ethnicity | .290 | 1 | .590 |
| Residuals |  | 1310 |  |

Supplemental Table 4. *Inferential statistics associated with ethnoracial background* × *WMH burden interaction effects on individual neurofilament light levels*

| Variables | *F* | df | *p* |
| --- | --- | --- | --- |
| MA ethnicity × WMH volume | 8.77 | 1 | .003 |
| Main Effects |  |  |  |
| Age | 184.36 | 1 | <.001 |
| Educational level | .168 | 1 | .682 |
| Sex | .226 | 1 | .635 |
| ICV | .022 | 1 | .881 |
| WMH volume | 42.34 | 1 | <.001 |
| Ethnicity | 5.23 | 1 | .022 |
| Residuals |  | 1298 |  |

Supplemental Table 5. *Inferential statistics associated with ethnoracial background* × *hypertension effects on individual neurofilament light levels controlling for Creatinine and Body Mass Index*

| Variables | *F* | df | *p* |
| --- | --- | --- | --- |
| MA ethnicity × Hypertension | 1.20 | 1 | 0.275 |
| Main Effects |  |  |  |
| Age | 256.68 | 1 | <.001 |
| Educational level | 5.79 | 1 | .016 |
| Sex | 51.54 | 1 | <.001 |
| Hypertension | 5.27 | 1 | .022 |
| Ethnicity | 5.53 | 1 | .019 |
| Creatinine | 241.46 | 1 | <.001 |
| BMI | 63.33 | 1 | <.001 |
| Residuals |  | 1296 |  |

Abbreviations: BMI = body mass index; MA = Mexican American

Supplemental Table 6. *Inferential statistics associated with ethnoracial background* × *cardiovascular disease effects on individual neurofilament light levels controlling for Creatinine and Body Mass Index*

| Variables | *F* | df | *p* |
| --- | --- | --- | --- |
| MA ethnicity × CVD | 11.10 | 1 | <.001 |
| Main Effects |  |  |  |
| Age | 282.124 | 1 | <.001 |
| Educational level | 8.42 | 1 | .004 |
| Sex | 52.62 | 1 | <.001 |
| CVD | 9.02 | 1 | .003 |
| Ethnicity | 1.94 | 1 | .164 |
| Creatinine | 251.20 | 1 | <.001 |
| BMI | 61.36 | 1 | <.001 |
| Residuals |  | 1296 |  |

Abbreviations: BMI = body mass index; CVD = cardiovascular disease; MA = Mexican American

Supplemental Table 7. *Inferential statistics associated with ethnoracial background* × *diabetes effects on individual neurofilament light levels controlling for Creatinine and Body Mass Index*

| Variables | *F* | df | *p* |
| --- | --- | --- | --- |
| MA ethnicity × Diabetes | 7.96 | 1 | .005 |
| Main Effects |  |  |  |
| Age | 284.16 | 1 | <.001 |
| Educational level | 2.46 | 1 | .117 |
| Sex | 49.75 | 1 | <.001 |
| Diabetes | 39.25 | 1 | <.001 |
| Ethnicity | 1.84 | 1 | .175 |
| Creatinine | 245.35 | 1 | <.001 |
| BMI | 79.23 | 1 | <.001 |
| Residuals |  | 1296 |  |

Abbreviations: BMI = body mass index; CVD = cardiovascular disease; MA = Mexican American

Supplemental Table 8. *Inferential statistics associated with ethnoracial background* × *White Matter Hyperintensity volume effects on individual neurofilament light levels controlling for Creatinine and Body Mass Index*

| Variables | *F* | df | *p* |
| --- | --- | --- | --- |
| MA ethnicity × WMH volume | 5.41 | 1 | .020 |
| Main Effects |  |  |  |
| Age | 125.99 | 1 | <.001 |
| Educational level | 4.72 | 1 | .030 |
| Sex | 48.56 | 1 | <.001 |
| ICV | .329 | 1 | .566 |
| WMH volume | 50.16 | 1 | <.001 |
| Ethnicity | 2.56 | 1 | .110 |
| Creatinine | 239.74 | 1 | <.001 |
| BMI | 74.01 | 1 | <.001 |
| Residuals |  | 1285 |  |

Abbreviations: BMI = body mass index; CVD = cardiovascular disease; ICV = intracranial volume; MA = Mexican American; WMH = white matter hyperintensity

Supplemental Table 9. *Inferential statistics associated with ethnoracial background* × *hypertension effects on individual neurofilament light levels controlling for Household Income and Acculturation*

| Variables | *F* | df | *p* |
| --- | --- | --- | --- |
| MA ethnicity × Hypertension | 2.70 | 1 | .101 |
| Main Effects |  |  |  |
| Age | 337.69 | 1 | <.001 |
| Educational level | .826 | 1 | .364 |
| Sex | .053 | 1 | .818 |
| Hypertension | 1.79 | 1 | .182 |
| Ethnicity | 2.27 | 1 | .132 |
| Income | 1.85 | 1 | .175 |
| SASH total score | 2.23 | 1 | .135 |
| Residuals |  | 1271 |  |

Abbreviations: BMI = body mass index; MA = Mexican American; SASH = Short Acculturation Scale for Hispanics

Supplemental Table 10. *Inferential statistics associated with ethnoracial background* × *cardiovascular disease effects on individual neurofilament light levels controlling for Household Income and Acculturation*

| Variables | *F* | df | *p* |
| --- | --- | --- | --- |
| MA ethnicity × CVD | 7.97 | 1 | .005 |
| Main Effects |  |  |  |
| Age | 351.08 | 1 | <.001 |
| Educational level | 1.28 | 1 | .258 |
| Sex | .081 | 1 | .776 |
| CVD | 5.11 | 1 | .024 |
| Ethnicity | 1.36 | 1 | .244 |
| Income | 1.97 | 1 | .161 |
| SASH total score | 1.84 | 1 | .175 |
| Residuals |  | 1271 |  |

Abbreviations: BMI = body mass index; CVD = cardiovascular disease; MA = Mexican American; SASH = Short Acculturation Scale for Hispanics

Supplemental Table 11. *Inferential statistics associated with ethnoracial background* × *diabetes effects on individual neurofilament light levels controlling for Household Income and Acculturation*

| Variables | *F* | df | *p* |
| --- | --- | --- | --- |
| MA ethnicity × Diabetes | 13.19 | 1 | <.001 |
| Main Effects |  |  |  |
| Age | 352.00 | 1 | <.001 |
| Educational level | .028 | 1 | .868 |
| Sex | .002 | 1 | .960 |
| Diabetes | 17.36 | 1 | <.001 |
| Ethnicity | .104 | 1 | .748 |
| Income | 1.34 | 1 | .248 |
| SASH total score | 1.36 | 1 | .243 |
| Residuals |  | 1271 |  |

Abbreviations: BMI = body mass index; MA = Mexican American; SASH = Short Acculturation Scale for Hispanics

Supplemental Table 12. *Inferential statistics associated with ethnoracial background* × *White Matter Hyperintensity volume effects on individual neurofilament light levels controlling for Household Income and Acculturation*

| Variables | *F* | df | *p* |
| --- | --- | --- | --- |
| MA ethnicity × WMH volume | 7.56 | 1 | .006 |
| Main Effects |  |  |  |
| Age | 179.36 | 1 | <.001 |
| Educational level | .338 | 1 | .561 |
| Sex | .499 | 1 | .480 |
| ICV | .025 | 1 | .875 |
| WMH volume | 35.91 | 1 | <.001 |
| Ethnicity | 1.32 | 1 | .252 |
| Income | .642 | 1 | .423 |
| SASH total score | 1.19 | 1 | .423 |
| Residuals |  | 1260 |  |

Abbreviations: BMI = body mass index; CVD = cardiovascular disease; ICV = intracranial volume; MA = Mexican American; SASH = Short Acculturation Scale for Hispanics; WMH = white matter hyperintensity

Supplemental Table 13. *Inferential statistics associated with ethnoracial background* × *White Matter Hyperintensity volume effects on individual left hippocampal volumes*

| Variables | *F* | df | *p* |
| --- | --- | --- | --- |
| MA ethnicity × WMH volume | .128 | 1 | .721 |
| Main Effects |  |  |  |
| Age | 6.81 | 1 | .009 |
| Educational level | .238 | 1 | .626 |
| Sex | .179 | 1 | .673 |
| ICV | 4.70 | 1 | .030 |
| WMH volume | 1.25 | 1 | .265 |
| Ethnicity | .047 | 1 | .829 |
| Residuals |  | 1298 |  |

Abbreviations: BMI = body mass index; CVD = cardiovascular disease; ICV = intracranial volume; MA = Mexican American

Supplemental Table 13. *Inferential statistics associated with ethnoracial background* × *White Matter Hyperintensity volume effects on individual right hippocampal volumes*

| Variables | *F* | df | *p* |
| --- | --- | --- | --- |
| MA ethnicity × WMH volume | .461 | 1 | .497 |
| Main Effects |  |  |  |
| Age | 6.81 | 1 | .009 |
| Educational level | .085 | 1 | .771 |
| Sex | .788 | 1 | .375 |
| ICV | 6.98 | 1 | .008 |
| WMH volume | .703 | 1 | .402 |
| Ethnicity | .212 | 1 | .645 |
| Residuals |  | 1298 |  |

Abbreviations: BMI = body mass index; CVD = cardiovascular disease; ICV = intracranial volume; MA = Mexican American
